# "*What-Where-Which*" Episodic Retrieval Requires Conscious Recollection and Is Promoted by Semantic Knowledge

**DOI:** 10.1371/journal.pone.0143767

**Published:** 2015-12-02

**Authors:** Anne-Lise Saive, Jean-Pierre Royet, Samuel Garcia, Marc Thévenet, Jane Plailly

**Affiliations:** Olfaction: from coding to memory team, Lyon Neuroscience Research Center, CNRS UMR 5292 - INSERM U1028, University Lyon1, Lyon, F-69366, France; University of Akron, UNITED STATES

## Abstract

Episodic memory is defined as the conscious retrieval of specific past events. Whether accurate episodic retrieval requires a recollective experience or if a feeling of knowing is sufficient remains unresolved. We recently devised an ecological approach to investigate the controlled cued-retrieval of episodes composed of unnamable odors (*What*) located spatially (*Where*) within a visual context (*Which context*). By combining the Remember/Know procedure with our laboratory-ecological approach in an original way, the present study demonstrated that the accurate odor-evoked retrieval of complex and multimodal episodes overwhelmingly required conscious recollection. A feeling of knowing, even when associated with a high level of confidence, was not sufficient to generate accurate episodic retrieval. Interestingly, we demonstrated that the recollection of accurate episodic memories was promoted by odor retrieval-cue familiarity and describability. In conclusion, our study suggested that semantic knowledge about retrieval-cues increased the recollection which is the state of awareness required for the accurate retrieval of complex episodic memories.

## Introduction

Episodic memory is defined as the conscious retrieval of personal experiences occurring within a specific context [1–3]. Although recognition memory is known to comprise at least two different states of awareness: *recollection* and *feeling of knowing* (or *familiarity*) [4–6], their role in rich and detailed episodic memory remains unresolved. The respective involvement of each of these two processes has been typically assessed using the Remember/Know (R/K) procedure based on participants’ introspection [4]. The participants must report whether they recognize items on the basis of *remembering* contextual details or associative information (*e*.*g*., an image, an emotion, a personal experience) or *knowing* that the item is familiar without any conscious recollection. These two states of awareness represent two different cognitive processes that rely on partially distinct neural substrates [7–9] and are affected differently by factors such as retention delay and intentional encoding [10]. Currently, associative or relational recall, such as that involved in episodic memory, is assumed to rely mainly on recollection, because only a “Remember” response would provide precise and specific information from the studied event [11–13]. The state of awareness underlying complex episodic memory has been experimentally tested only twice, with inconsistent results [14,15]. The objective of the present study was to examine the state of awareness associated with the retrieval of rich multidimensional episodes or, in other words, to determine whether accurate episodic retrieval requires a recollective experience or if a feeling of knowing is sufficient.

Researchers either test autobiographical memory by interrogating participants about real-life memories encoded in their past [16–20] or they test the memorization of artificial episodes created in the laboratory using recognition tasks [21–25]. To combine the richness of real-life memories investigated through an ecological approach and the control of memory accuracy that is possible in a laboratory-based approach, we recently developed a novel *laboratory-ecological* task [26–28]. This approach allows for the controlled study of the cued-retrieval of trial-unique and complex multimodal episodes composed of unnamable odors (*What*) located spatially (*Where*) within a visual context (*Which context*).

In the current study, we combined the R/K procedure with our laboratory-ecological approach [26,28] in an original way to investigate (*i*) the respective requirement of odor recollection and odor familiarity in the accurate retrieval of odor-evoked episodic memories and (*ii*) the properties of the odor retrieval-cues and the breathing modulations accompanying each of these states of awareness.

## Materials and Methods

### Ethics statement

The study has been approved by the local Institutional Review Board in accordance with French regulations for biomedical experiments with healthy volunteers (Ethical Committee of CPP Sud-Est IV: CPP 11/007, ID RCB: 2010-A-01529-30). The study was conducted in accordance with the Declaration of Helsinki. The participants provided written informed consent.

### Participants

Twenty-three healthy participants [15 women, age: 21.9 ± 2.02 years (mean ± standard deviation)] consented to participate in the experiment and received 30 euros in compensation. All participants were right-handed and reported normal senses of smell and no visual impairments.

### Stimuli and materials

#### Stimuli

Eighteen odorants, of which most have been used in earlier studies [26,28], were selected based on their distinctiveness and relatively low identifiability and familiarity and were subdivided into two sets (Sets 1 and 2) of nine odorants each. Set 1 consisted of butanol, carrot, cis-3-hexenyl salicylate, dihydromyrcenol, heptanon, methyl octine carbonate, musk, rosemarel and stemone. Set 2 consisted of 9-decen-1-ol, basil, birch oil, citronellol, ethyl acetyl acetate, linalyl acetate, rose oxide, tobacco and tomato.

The odorants were presented using a twenty-channel computer-controlled olfactometer that was connected to the nostrils. The participants were requested to breathe normally and avoid sniffing behaviors. Each participant’s respiratory signal was acquired using a nasal cannula and was used to trigger the odor stimulation through an airflow sensor. The airflow rate was set at 3 l/min, and the odorants were delivered over 4 s.

**Fig 1:**
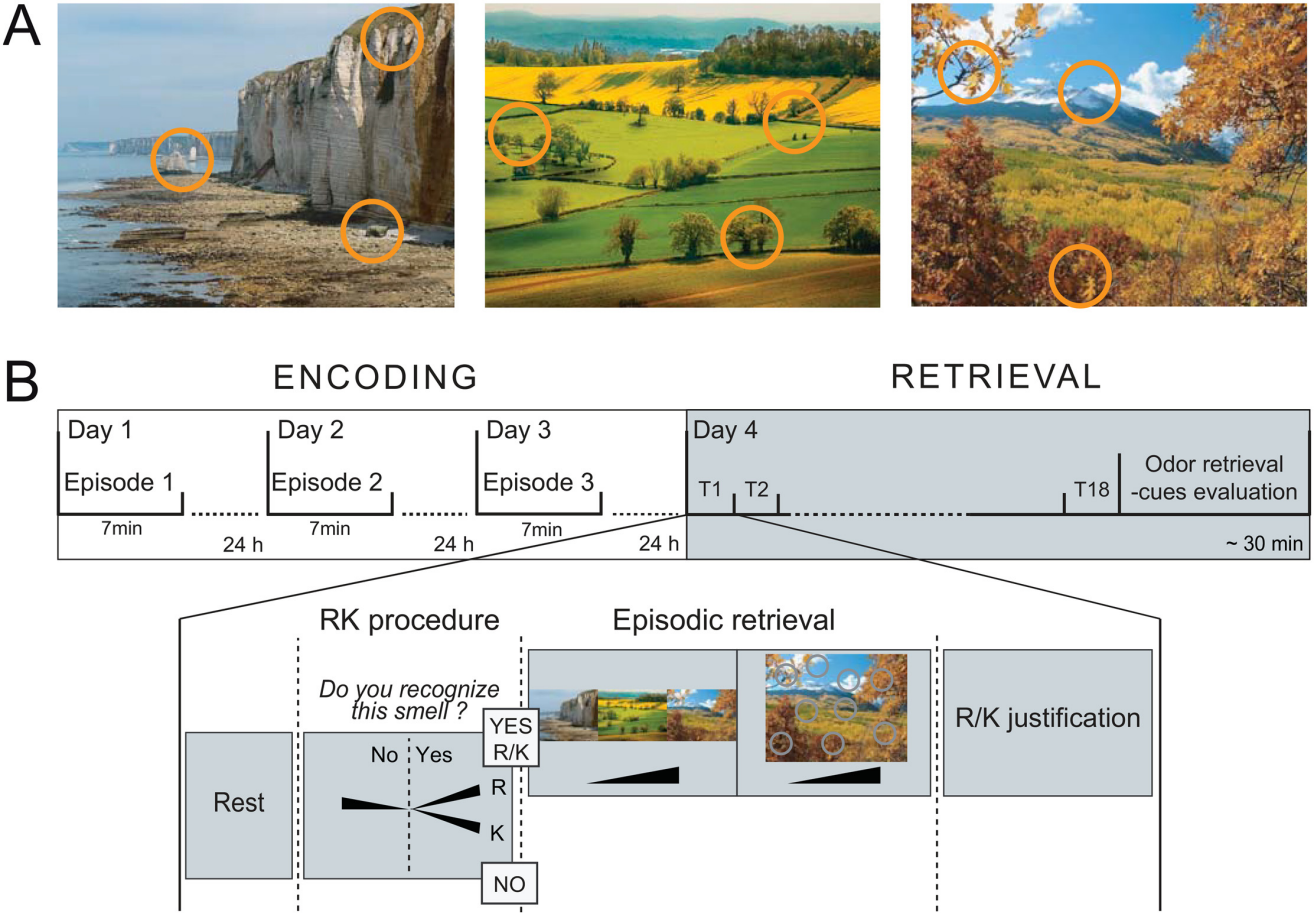
Episodic memory task design and Remember/Know procedure. (A) The three spatio-contextual environments of the episodes. Orange circles represent the spatial locations associated with an odor. (B) The temporal course of the encoding and retrieval sessions, with an example of a retrieval trial. During the encoding, the participants discovered one episode per day over three days. On the fourth day, the memory of the episodes was evaluated using an odor recognition task (R/K procedure) followed for the R and K trials by an episodic retrieval task. K, Know; R, Remember; T, Trial.

Three landscape pictures presented in full-screen view constituted the visual contexts (a coastal cliff, a countryside and a mountain landscape). Three orange circles in each image symbolized the three spatial locations associated with an odor (Fig 1A).

#### Multidimensional episodes

Three multidimensional episodes were created. Each episode was composed of three odors (*What*) associated with specific locations (*Where*) within a visual context (*Which context*). The contexts, spatial locations and odors differed between episodes. To limit associative semantic processes, the odors, spatial locations and visual contexts were arbitrarily linked. An in-house LabView software (National Instruments^®^, Austin, TX, USA) connected to the olfactometer controlled the presentation of odors, pictures and circles and recorded the participants’ responses and breathing throughout the experiment. The participants used a trackball to interact with the software. When they clicked on a circle, the odor stimulus was delivered at the beginning of the subsequent expiration, allowing the odor to be perceived at the beginning of the next inspiration. The volume, amplitude and duration of each inspiratory cycle were measured, and the respiratory frequency was calculated.

### Experimental Procedure

The experimental procedure consisted of four sessions performed over the course of four successive days. The encoding took place during the first three sessions, and the retrieval occurred on the fourth session (Fig 1B). A full night of sleep followed each of the encoding sessions to promote consolidation and to reduce interference [29,30]. Participants completed the four sessions at the same time of day to limit the differential influences of internal states (hunger, satiety) on olfactory and cognitive processes between sessions [31,32].

#### Encoding session

During the encoding, the participants freely discovered one episode per day for 7 min (Fig 1B). They were asked to explore all dimensions of the episode as much as possible by paying attention to the background picture, the circles superimposed on this background, and the odors that were delivered when clicking on the circles. No memorization instruction was given, thereby ensuring free encoding in a manner similar to what occurs in real-life situations. The participants were informed that they would be questioned about their perception of the episodes on the fourth day (see S1 Fig for the complete participant encoding instructions). The order of the three episodes was counterbalanced between participants.

#### Retrieval session

To investigate the states of awareness accompanying episodic retrieval, we adapted the retrieval procedure of Saive et al. [28] to allow for a one-step R/K procedure (Fig 1B). The retrieval session consisted of one block of 18 trials, corresponding to the presentation of 9 target odors randomly intermixed with 9 distractor odors. The use of the Set 1 or Set 2 odorants as targets or distractors was counterbalanced between the participants.

***Odor recognition task***. For each odor, the participants had to decide if they recognized the smell or not (“Yes” or “No” response). If they did, they had to determine whether they “remembered” the odor from the studied episodes (“R” response) or whether they just “knew” that it was previously perceived (“K” response). The R response represented a conscious recollection of some specific contextual information associated with the odor during the encoding (*e*.*g*., a picture, a personal experience), whereas the K response represented a feeling of knowing in the absence of conscious recollection of the odor’s previous presentation. When giving their responses, the participants were asked to simultaneously rate their subjective level of confidence using a slider on a non-graduated scale, a procedure adapted from Ingram et al. [33]. The distinction between the R/K responses and the confidence strength was emphasized, and the participants were told that both recollection and familiarity can vary in strength [33,34]. Detailed instructions and examples explaining the differences between R and K judgments were given to the participants, and their comprehension was checked before the retrieval session (see S2 Fig for the complete participant retrieval instructions).

***Episodic retrieval task***. Following the R and K responses, the participants were asked to retrieve the entire episode associated with the odor by choosing both a visual context (one of the three pictures) and a location (one of the nine circles superimposed on the chosen picture). They also had to rate their level of confidence for both the picture and the location using a slider on a non-graduated scale. A response was considered correct if the participants selected both the accurate context and the specific location previously associated with the odor during the encoding. If the participants rejected the odor (“No” response), they rested until the next trial for 3 s. All the retrieval steps were self-paced. At the end of each trial, the participants were asked to explain their R/K responses. These justifications were used to correct for misattributions if necessary.

***Odor retrieval-cue evaluation***. At the end of the retrieval session, the participants were asked to rate the odors in terms of pleasantness, intensity, and familiarity using non-graduated scales and to describe them if possible.

### Data analysis

During retrieval, recognition memory performance was assessed using parameters from the signal detection theory [35]. Four response categories were defined: Hit and Miss corresponded to the accurate recognition and the inaccurate rejection of target odors, respectively, while correct rejection (CR) and false alarm (FA) corresponded to the accurate rejection and the inaccurate recognition of distractor odors, respectively. A memory score (*d’*_*L*_) reflecting the participant’s ability to discriminate between the target and distractor odors was calculated [36] (see S3 Fig for detailed calculations).

In the episodic retrieval test, we defined four types of responses depending on the accuracy of the memory triggered by accurate odor recognition (Hit). When the participants correctly recognized the target odors, they could accurately remember both the location and the context (WWW), the location only (WWhere), the context only (WWhich) or be mistaken about both dimensions (What). The theoretical proportions of these episodic combinations were 0.019, 0.037, 0.148 and 0.296, respectively (see S4 Fig for detailed calculations). The WWhere response occurred only once for one participant and was therefore excluded from the analyses. The number of R/K responses was calculated for the response categories (FA, Hit, WWW, WWhich and What), and the subscripts R or K were added to indicate the corresponding conditions (*e*.*g*., *FA*_*R*_, *FA*_*K*,_ *WWW*_*R*,_ *WWW*_*K*_). The recollection score (*Rec*; Jacoby, 1991) reflecting the proportion of accurate recollection was calculated as follows:

$$Rec=\frac{Hit_{R}}{Hit}-\frac{FA_{R}}{FA},$$

where *Hit*_*R*_ and *FA*_*R*_ represent the numbers of accurate and inaccurate recollections, respectively. With the probability of randomly giving an R, K or No response being equal, the calculation of theoretical proportions of the R/K episodic combinations was 0.006 for WWW (*i*.*e*., 0.019/3), 0.049 for WWhich (*i*.*e*., 0.148/3), and 0.099 for What (*i*.*e*., 0.296/3).

The confidence evaluations were *a posteriori* transformed into values ranging from 0 to 1. The confidence for the episodic retrieval responses was defined as the mean of the context and location confidences. The breathing parameters (*i*.*e*., the volume, amplitude and duration of the inspiratory cycles and the respiratory frequency) were extracted between the time at which the odor was delivered and the R/K responses. The means of these variables were determined for all R/K responses.

The odor retrieval-cue pleasantness, intensity, and familiarity ratings were *a posteriori* transformed into values ranging from 0 to 10. For familiarity, a value of 5 represented the boundary between unfamiliar and familiar odors. Odors rated as 5 were assigned to both groups. For pleasantness, values below 4 represented unpleasant odors, values between 4 and 6 represented neutral odors and values above 6 represented pleasant odors. The odor descriptions were transformed into scores of 1 and 0 based on whether the participants provided any description (*e*.*g*., medicine, spicy, sour, garden, soft, subtle, stinky cheese, oppressive) or not (there were no veridical labels because odors were uncommon odors).

### Statistical analysis

The main effects of the factors and interactions were determined using repeated measures ANOVAs for the variable “Number of responses.” Confidence evaluations and breathing measures for odor and context were analyzed using two-way ANOVAs to allow for statistical comparisons even in the absence of some conditions for some participants. ANOVAs were followed by *post-hoc* bilateral Student’s *t*-tests if the main effects and/or interactions were significant. The “Proportions of responses” were compared to the respective theoretical proportions using Student’s *t*-tests. The effects were considered significant at *p* < 0.05. Statistical analyses were performed using Statistica (StatSoft^®^, Tulsa, OK, USA).

## Results

### Odor retrieval-cue evaluation

On average, the odorants were perceived as relatively neutral (4.83 ± 1.13, range: 3.36–6.74), moderately intense (6.22 ± 0.76, range: 4.14–7.24), with all odors being perceivable, moderately familiar (5.10 ± 1.25, range: 3.76–7.10), and moderately describable (0.63 ± 0.43).

### Memory performance

#### Odor recognition

The participants were very proficient in recognizing old odors and rejecting new ones, as indicated by the high memory score (*d*’_*L*_ = 3.30 ± 1.37) and numbers of correct responses (Hit = 7.57 ± 1.44 of 9 target odors; CR = 7.52 ± 1.16 of 9 distractor odors), which were far above the chance level (*t*_(22)_’s > 10.21, *p*_*s*_ < 0.001). This pattern of behavioral performance replicates our previous results [26,28] and indicates that the addition of the R/K procedure did not alter the recognition performance.

**Fig 2:**
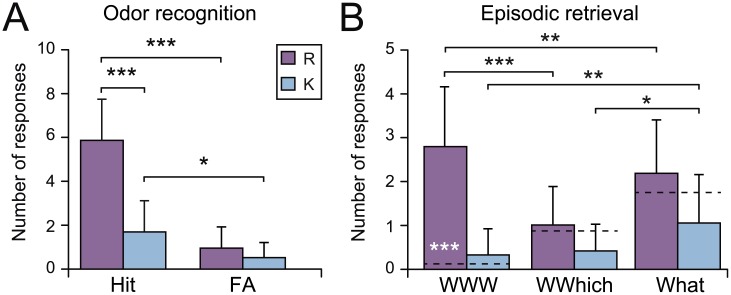
Memory performance. Mean numbers of R/K responses as a function of A) the accurate (Hit) and inaccurate (FA) odor recognition, and B) the episodic retrieval responses (WWW, WWhich and What). The dashed horizontal lines indicate the chance levels of the WWW, WWhich and What responses. Vertical bars represent the SD; in black, * *p* < 0.05, ** *p* < 0.01, *** *p* < 0.001; in white, *** *p* < 0.001 above chance level.

When recognizing an odor, the participants made a simultaneous R/K judgment (Fig 2A). A two-way R/K judgment x accuracy (Hit/FA) ANOVA revealed a significant effect of the R/K response [*F*_(1, 22)_ = 42.07, *p* < 0.001] due to a higher number of R responses (6.83 ± 2.33) than K responses (2.65 ± 4.47). A significant R/K-by-accuracy interaction [*F*_(1, 22)_ = 32.39, *p* < 0.001] revealed that the pattern of R/K responses was obtained for accurate recognitions (*Hit*_*R*_ *vs*. *Hit*_*K*_; *p* < 0.001) but not for inaccurate recognitions (*FA*_*R*_ *vs*. *FA*_*K*_; *p* > 0.35). Accurate recognitions were preferentially associated with R responses, whereas false memories were indifferently associated with R or K responses. Considering R responses only, the number of accurate recognitions (*Hit*_*R*_) was higher than the number of inaccurate recognitions (*FA*_*R*_) (*p* < 0.001). These results were consistent with a recollection score higher than the chance value of zero (*Rec =* 0.55 ± 0.22; *t*_(22)_ = 12.07, *p* < 0.001) and reflected that the accurate odor recognition was mainly related to recollective experience. Considering K responses only, the number of Hit was higher than the number of FA (*Hit*_*K*_ *vs*. *FA*_*K*_; *p* = 0.019), indicating that the feeling of knowing was sufficient to achieve odor retrieval-cue recognition.

#### Episodic retrieval

Following the accurate recognition of previously perceived odors (Hit), the participants were asked to retrieve the contextual and spatial dimensions of the episode associated with the odor. The numbers of accurate and inaccurate episodic retrieval responses (WWW: 3.09 ± 1.31; What: 3.09 ± 1.53) were significantly higher than the number of incomplete retrieval responses (WWhich: 1.35 ± 0.88) [*F*_(2, 44)_ = 11.14, *p* < 0.001; *post-hocs*, *p*_*s*_ < 0.001]. Moreover, the number of accurate episodic retrieval responses (WWW) was far above the chance level [*t*_(22)_ = 10.68, *p* < 0.001], whereas the numbers of incomplete (WWhich) and inaccurate (What) episodic retrieval responses were not significantly different from chance [*t*_(22)_’s < 1.31, *p*_*s*_ > 0.20]. Thus, either the participants retrieved complete episodes triggered by accurate odor recognition or they answered randomly.

The accuracy of the episodic retrieval triggered by odor recognitions associated with either R or K responses was examined (Fig 2B). A two-way R/K x Episodic ANOVA showed a significant interaction between both factors [*F*_(2, 44)_ = 14.62, *p* < 0.001]. A higher number of R than K responses was observed in the three episodic conditions (WWW, *p* < 0.001; WWhich, *p* = 0.034; What, *p* < 0.001). Higher numbers of R responses for WWW and K responses for What were found compared to the other two episodic conditions (R responses: WWhich, *p* < 0.001 and What, *p* = 0.010; K responses: WWW, *p* = 0.010 and WWhich, *p* = 0.023). Additionally, the number of R responses was significantly higher than the number of theoretical random responses in the WWW condition (*t*_(22)_ = 9.76, *p* < 0.001) but was not significantly different from chance in the WWhich and What conditions [*t*_(22)_ = 0.39 and *t*_(22)_ = 1.27, *p*_*s*_ > 0.20]. The numbers of K responses were not significantly different or were significantly lower than the corresponding numbers of random responses in the WWW [*t*_(22)_ = 1.66, *p* > 0.09], WWhich and What [*t*_(22)_ = -4.09 and *t*_(22)_ = -3.57, *p*_*s*_ < 0.001] conditions. Briefly, the complete and accurate episodic retrieval was observed only when the participants accurately remembered the information (WWW_R_) but not when their responses were based on a feeling of knowing (WWW_K_).

#### Confidence evaluations

**Fig 3:**
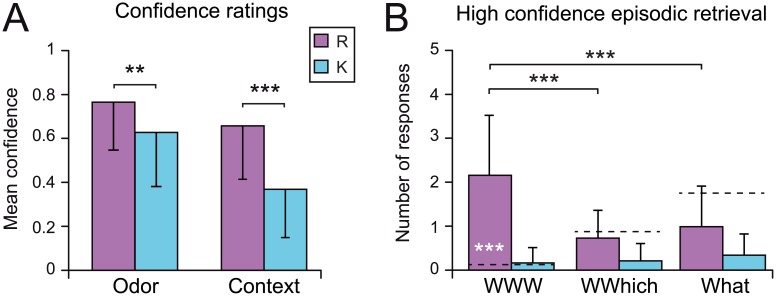
Confidence ratings and episodic retrieval. A) Mean levels of confidence of the odor recognition response (Odor) and the visuospatial context retrieval (Context) given the R/K responses. B) Mean numbers of R/K responses in the high confidence episodic retrieval. Vertical bars represent the SD; in black, ** *p* < 0.01, *** *p* < 0.001; in white, *** *p* < 0.001 above chance level.

We examined the confidence of the odor recognition and of the visuospatial context retrieval and tested whether it differed according to the R/K and episodic retrieval (WWW, WWhich, What) responses. For the odor recognition, a two-way R/K x Episodic ANOVA revealed a significant effect of R/K responses [*F*_(1, 83)_ = 8.67, *p* = 0.004] (Fig 3A, Odor) but no significant effect of Episodic responses [*F*_(2, 83)_ = 1.54, *p* > 0.21] and no significant interaction between both factors [*F*_(2, 83)_ = 0.89, *p* > 0.41]. For the visuospatial context retrieval, we observed a significant effect of the R/K factor [*F*_(1, 83)_ = 24.85, *p* < 0.001] (Fig 3A, Context) but no significant effect of the Episodic factor [*F*_(2, 83)_ = 2.25, *p* > 0.10] and no significant interaction between both factors [*F*_(2, 83)_ = 0.16, *p* > 0.84]. Briefly, the confidence of participants in their responses during both odor recognition and visuospatial context retrieval was higher when they experienced recollection compared to a feeling of knowing.

#### High confidence responses

To disentangle the R/K responses from the confidence judgments, we considered only the high-confidence R/K responses, in which odor confidence was equal or superior to the mean odor confidence (0.73 ± 0.19) (Fig 3B). First, we confirmed that proportions of high-confidence accurate odor recognitions (Hit) compared to the total number of recognitions (Hit + FA) were similar when considering R responses [*Hit*_*R*_/(*Hit*_*R*_+*FA*_*R*_) = 0.91 ±0.15] or K responses [*Hit*_*K*_/(*Hit*_*K*_+*FA*_*K*_) = 0.73 ± 0.44; *F*_(1, 34)_ = 3.15, *p* > 0.08]. This result indicated that odor recognition accuracy was not related to R/K responses in high confidence responses. Second, a two-way R/K x Episodic ANOVA on the number of high-confidence responses revealed similar results to those obtained by including all confidence responses (see 3.2.2 Episodic Retrieval) [R/K: *F*(_1, 22_) = 43.45, *p* < 0.001; Episodic: *F*(_2, 44_) = 11.93, *p* < 0.001; R/K-by-Episodic: *F*(_2, 44_) = 14.78, *p* < 0.001]. Consistently, the number of WWW_R_ was significantly above chance (*t*_(22)_ = 10.46, *p* < 0.001) and the number of WWW_K_ responses did not differ from the number of random responses (*t*_(22)_ = 1.39, *p* > 0.19). In other words, a feeling of knowing, even when associated with a high level of confidence, was not sufficient to generate accurate episodic retrieval.

#### Retrieval-cue properties and breathing modulations

**Fig 4:**
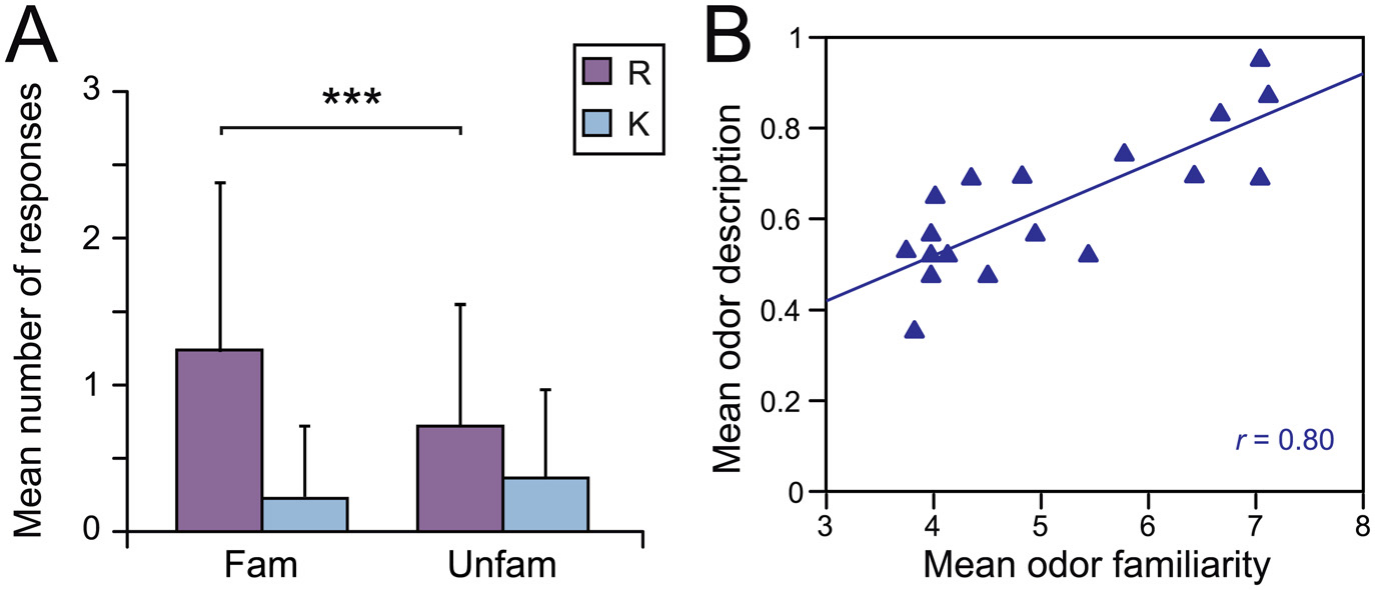
Odor familiarity and describability. A) Mean numbers of R/K responses as a function of familiarity of odors. Fam, Familiar; Unfam, Unfamiliar; Vertical bars represent the SD; ** *p* < 0.01, *** *p* < 0.001. B) Correlation between familiarity and describability of odors.

***Familiarity*.** We examined whether the R/K and the Episodic retrieval (WWW, WWhich, What) responses varied as a function of the familiarity of odor retrieval-cues (Unfamiliar, Familiar). A two-way ANOVA revealed that the familiarity of odors significantly influenced the R/K responses [*F*_(1, 22)_ = 7.33, *p* = 0.002], but not the Episodic retrieval [*F*_(2, 44)_ = 0.81, *p* > 0.44], and no significant interaction between these factors was found [*F*_(2, 44)_ = 2.53, *p* > 0.08]. The familiar odors generated more recollective experience than the unfamiliar odors (Fig 4A). Furthermore, the odors’ familiarity was significantly positively correlated with the odors’ describability [*r* = 0.80, *t*_(1, 18)_ = 5.35, *p* < 0.001, Pearson’s test] (Fig 4B). The more familiar the odors, the more they were described by the participants.

***Pleasantness***. A two-way R/K x Episodic ANOVA showed that the pleasantness of the odor retrieval-cues (Pleasant, Neutral, Unpleasant) had no significant effect on R/K [*F*_(2, 44)_ = 0.04, *p* > 0.95] and Episodic retrieval [*F*_(4, 88)_ = 0.62, *p* > 0.64] responses. No significant interaction between these factors [*F*_(4, 88)_ = 0.41, *p* > 0.79] was found.

***Breathing***. We explored whether the volumes, durations, amplitudes and frequencies of the inspirations measured during retrieval varied as a function of R/K and Episodic responses (WWW, WWhich, What). Two-way ANOVAs revealed that the inspiration measures did not vary as a function of R/K responses and Episodic retrieval responses [*F*_(1, 83)_’s < 2.15, *p*_*s*_ > 0.13 and *F*_(2, 83)_’s < 1.50, *p*_*s*_ > 0.22, respectively], and no significant interactions between these factors were found [*F*_(2, 83)_’s < 0.36, *p*_*s*_ > 0.69].

## Discussion

The present study examined the involvement of recollection and feeling of knowing in complex episodic memory retrieval by investigating the odor-triggered retrieval of rich and close-to-real-life episodes. Although these two states of awareness supported accurate recognition memory of the odor, the retrieval of the full episode overwhelmingly required recollection. The feeling of knowing was insufficient, even when only high-level confidence responses were concerned. Interestingly, recollective experience was promoted by odor familiarity and describability. Higher semantic knowledge about odor retrieval-cues promoted the conscious recollection that was required for the retrieval of accurate episodic memories.

Only two studies have already investigated the requirement of recollection in cued-retrieval of complex episodic memories. First, Holland and Smulders [37] developed a “*What-Where-When*” memory task, in which the participants had to remember the locations of a room in which they hid coins of various values on two consecutive days. No R/K procedure was conducted, but the participants reported using a mental time travel strategy to recall the spatial locations that suggested a recollective experience. Second, Easton et al. [15] created a task in which the participants were questioned about two episodes immediately after their explicit encoding. By choosing one of two answer options, the participants answered questions about either the order of the power-point slide (*When*) or the visual background (*Which*) associated with an abstract symbol (*What*) located in a particular spot on the screen (*Where*). In contrast to the “*What-Where-When*” memory task, in which familiarity (*i*.*e*., the strength of the memory trace) was sufficient, the “*What-Where-Which*” memory task was accurately performed only using recollection. Our experiment confirmed the necessity of recollection in the accurate retrieval of complex episodes and validated the Remember/Know procedure in an ecological setting. Importantly, when the participants accurately recognized the odors, either they retrieved complete and accurate episodes or they answered randomly. This result is in agreement with the idea we previously proposed that when the binding between the odors and the spatio-contextual dimensions of the episodes is successful, the odor recognition and the episodic retrieval process collapsed into a unique memory process [28]. Together, these findings suggest that when accurate the retrieval of complex episodes is underpinned by one unique memory process involving recollection.

The cross-modal nature and the low semantic content of the episodes could explain why their retrieval was based on conscious recollection. In our task, the participants were asked to retrieve specific episodes comprised of dimensions of various modalities. The associations were arbitrary and included unfamiliar and hard-to-identify odors, limiting the use of semantic links to bind episode dimensions together. In associative recognition studies, the contribution of recollection and familiarity depends on the semantic relationships between items and on the cross-modality of the associations. Recollection is required to recognize arbitrary associations between items [12,38]. On the one hand, between-item and between-domain associations, that are associations respectively formed between various types of items or modalities (*e*.*g*., faces and voices), rely on recollection more than intra-item or within-domain associations [39,40]. On the other hand, familiarity is greater for intra-item associations (*e*.*g*., paired words forming a compound word) than for within-domain associations (*e*.*g*., unrelated words) [41]. Overall, in associative recognition studies, the more distant the items of the associations in terms of modality or semantic relationships, the more their recognition requires recollection. Our episodic memory study extended these observations by demonstrating that the more distant the dimensions of the episodes in terms of modality or semantic relationships, the more their retrieval requires recollection.

Even if odors were hardly identifiable, our results revealed that the semantic knowledge about the odor retrieval-cues favored the conscious recollection of the entire complex episode. The semantic knowledge was figured by the odor familiarity and describability. Familiar odors have been shown to evoke semantic information that enables their identification [42] and to generate greater recollective experience in recognition memory [43]. Here, odor familiarity, which was intrinsically related to the verbal describability of the odor, triggered more episodic retrieval based on remembering than on the feeling of knowing. Our findings confirmed the widespread hypothesis that semantic knowledge increases conscious recollection but not the feeling of knowing in the remembrance of episodic memories (for a review [10], but [44]). How does semantic knowledge promote recollection? First, when retrieving experienced events, we piece together our memory of the items (persons, objects) and the context in which we encoded these items. The context of the events is part of a lifetime period and contains semantic and conceptual information (*e*.*g*., locations, dates, relationships) [45,46]. Here, our results argued for the idea that the feeling of familiarity enhances the description of the odors even if they were mostly limited to an adjective or an olfactory note (*e*.*g*., minty, spicy). Associating an odor with prior semantic knowledge seems to promote its recognition and the recollection of contextual details. Second, processing information in relation to the self is also known to increase the recollective processes during memory retrieval [47,48]. We could suppose that the familiarity evoked by previously encountered odors enhances self-reference processes during encoding and therefore increases recollection during episodic retrieval.

Finally, the influence of the confidence level accompanying recollection and familiarity requires further discussion. Many studies examining the subjective processes accompanying recognition memory have shown that, on average, recollection is associated with high confidence responses, whereas a feeling of knowing is associated with low confidence responses [49–52]. During odor recognition and episodic retrieval, our results were consistent with these observations and corroborated the association of recollection with a higher level of confidence than with the feeling of knowing. Thus, it could be claimed that the impossibility of the feeling of knowing to support accurate episodic retrieval is related to the low confidence the participants had in their responses rather than to their state of awareness. However, even in restricting our analysis to responses with high confidence values, our observation of same results argued for the requirement of recollection in retrieving episodic memories.

## Conclusion

Briefly, our results showed that cross-modal “*What*-*Where*-*Which”* accurate odor-evoked episodic retrieval overwhelmingly relied on recollective processes. Additionally, the feeling of familiarity evoked by odor retrieval-cues increased the recollective experience, leading to the accurate remembering of the odor and its associated dimensions. Familiar odors benefited from greater semantic knowledge than unfamiliar odors, which induced a stronger episodic memory trace and a greater recollection during episodic retrieval. Together, our study suggested that semantic access to episodic memory promoted by odor retrieval-cue familiarity increased recollection, which is the state of awareness required for accurately retrieving complex episodic memories.

## Supporting Information

S1 Fig Encoding instructions. (DOCX)

S2 Fig Retrieval instructions. (DOCX)

S3 Fig Calculation of d’_L._
(DOCX)

S4 Fig Calculation of theoretical proportions of episodic combinations. (DOCX)
