# Supplementary material for: "What-Where-Which" Episodic Retrieval Requires Conscious Recollection and Is Promoted by Semantic Knowledge
Source: PLoS One. 2015 Dec 2;10(12):e0143767. doi: 10.1371/journal.pone.0143767 (PMC4668091; doi:10.1371/journal.pone.0143767)
Supplement: S1 Fig — (DOCX) [file pone.0143767.s001.docx]

**S1 Appendix. Encoding instructions**

“*During the first three sessions, a different and complex environment is presented to you each day for 7 minutes. Your goal is to explore all dimensions of each environment as much as possible. On the fourth day, we will test the perception that you had of these environments*.”

“*You are free to explore the environments at your own pace by paying attention to the background picture, the circles superimposed on this background, and the odors that are delivered when clicking on the circles. Each circle is associated with a different odor but the same circle is always associated with the same odor. The pictures, circles’ positions and odors are different every day. We ask you to breathe normally and constantly and to avoid sniffing when you click on the circles. When you click on a circle, the odor stimulus is delivered at the beginning of your subsequent expiration to enable perception of the odor at the beginning of your next inspiration. Do not pay attention to this delay and continue to breathe normally. You can click as much as you want on the circles to smell the odors but you need to respect a certain delay between two clicks to limit the saturation of your smell capacities. To click on the circles, use the trackball in front of you.”*

*“Now, we are going to run a test trial to be sure you understand everything. Do not hesitate to ask if you have any questions.”*
