# Supplementary material for: "What-Where-Which" Episodic Retrieval Requires Conscious Recollection and Is Promoted by Semantic Knowledge
Source: PLoS One. 2015 Dec 2;10(12):e0143767. doi: 10.1371/journal.pone.0143767 (PMC4668091; doi:10.1371/journal.pone.0143767)
Supplement: S2 Fig — (DOCX) [file pone.0143767.s002.docx]

**S2 Appendix. Retrieval instructions**

*“The goal of this session is to test your memory of the three environments you discovered in these last three days. We are going to present you with odors. For each odor, you have to determine whether or not you have already smelled it in the experiment in the last three days. When you recognize an odor as having already been smelled, we ask you to retrieve the context that was associated with it, which is the background picture and the location of the orange circle you clicked on to smell it.”*

*“In more detail, for each odor, you have to determine whether you ‘remember’ the odor from the previous environments, whether you only ‘know’ that the odor has been previously encountered in one of the last three days, or whether you have never smelled the odor during the experiment. You might answer ‘Remember’ if the recognition is associated with the recovery of associated information, even if it was not necessarily related to the experiment (e.g., emotion, personal experience). If you only know that the odor is familiar and has already been smelled in the experiment, but you do not remember anything else, you might answer “Know”. To clarify this distinction, here is an example: When someone asks you “what’s your name?” you know your name but most of the time no other related information comes to your mind when you answer. This is a Know response. However, if someone asks “what’s the name of the last movie you saw?” when you retrieve this information, you can remember elements associated with the movie such as images, the story, comments you made with your friends after, and so on. This is a Remember response. Is the difference clear to you? For all your Remember/Know responses, we ask you to justify your answer. Thus, for the Remember responses, you have to detail the information you retrieved. Whatever your response, Remember, Know or No, you have to click on a non-graduated scale to simultaneously rate your confidence. The more confident you are in your answer, the more you use the wide part of the scale. To clarify, Remember and Know responses do not reflect the confidence you have in your answer. You can be poorly confident of a Remember response, such as the name of the last movie you saw, and very confident of a Know response, such as your name.”*

*“Following the Remember and Know responses, you are asked to retrieve the entire episode associated with the odor by choosing both a visual context and a location. To do so, you must first select one of the three background pictures. This picture will then be displayed full-screen with the nine circles superimposed on it, and you must select one of those circles. When reconstructing the episode, you are asked to rate your confidence of both the picture and the location using a slider on a non-graduated scale. If you answer that you have never smelled the odor, you will rest until the next trial for 3 s.*

*“The retrieval is self-paced so feel free to take your time. Now, we are going to run a test trial to show you the various steps of the experiment and to be sure you understand everything. Do not hesitate to ask if you have any questions.”*
