# Supplementary material for: "What-Where-Which" Episodic Retrieval Requires Conscious Recollection and Is Promoted by Semantic Knowledge
Source: PLoS One. 2015 Dec 2;10(12):e0143767. doi: 10.1371/journal.pone.0143767 (PMC4668091; doi:10.1371/journal.pone.0143767)
Supplement: S3 Fig — (DOCX) [file pone.0143767.s003.docx]

**S3 Appendix. Calculation of d’_L_**

In the framework of the signal-detection theory, a memory score (*d’_L_*) reflected the participant’s ability to discriminate between the target and distractor items. This score was determined from the Hit and FA scores and was calculated as follows:

$$d'_{L}=\ln\frac{HR(1-FR)}{FR(1-HR)}$$

where *HR* represents the Hit rate [(Hit + 0.5) / (*N_t_* + 1)], *FR* represents the false alarm rate [(FA + 0.5) / (*N_d_* + 1)] and *N_t_* and *N_d_* represent the number of target and distractor odors, respectively, for which the participants provided an answer. Memory scores may be good or poor (positive or negative values, respectively).
