# Supplementary material for: "What-Where-Which" Episodic Retrieval Requires Conscious Recollection and Is Promoted by Semantic Knowledge
Source: PLoS One. 2015 Dec 2;10(12):e0143767. doi: 10.1371/journal.pone.0143767 (PMC4668091; doi:10.1371/journal.pone.0143767)
Supplement: S4 Fig — (DOCX) [file pone.0143767.s004.docx]

**S4 Appendix. Calculation of theoretical proportions of episodic combinations**

The theoretical proportions of the episodic combinations resulting from responses given randomly were 0.019 for WWW [1 response (“*Yes/No*”) out of 2 * 1 context out of 3 * 1 location out of 9], 0.148 for WWhich [1 response (“*Yes/No*”) out of 2 * 1 context out of 3 * 8 locations out of 9], 0.037 for WWhere [1 response (“*Yes/No*”) out of 2 * 2 contexts out of 3 * 1 location out of 9] and 0.296 for What [1 response (“*Yes/No*”) out of 2 * 2 contexts out of 3 * 8 locations out of 9].
